# Physical Activity during Weekdays and Weekends in Persons with Multiple Sclerosis

**DOI:** 10.3390/s21113617

**Published:** 2021-05-22

**Authors:** Yoshimasa Sagawa, Eric Watelain, Thierry Moulin, Pierre Decavel

**Affiliations:** 1 Laboratory of Clinical Functional Exploration of Movement, Department of Physical Medicine and Rehabilitation, University Hospital of Besançon, F-25000 Besançon, France; Pierre.Decavel@h-fr.ch; 2 Laboratory of Integrative Research in Neurosciences and Congnitive Psycology EA481, Faculty of Medicine and Health Sciences, Bourgogne Franche-Comte University, F-25000 Besançon, France; thierry.moulin@univ-fcomte.fr; 3 Laboratory IAPS, UR 20172320F, Faculty of Sport Science, Toulon University, F-83041 Toulon, France; eric.watelain@univ-tln.fr; 4 Department of Neurology, University Hospital of Besançon, F-25000 Besançon, France

**Keywords:** multiple sclerosis, accelerometer, sensors, physical activity, gait, exercise

## Abstract

The assessment of the functional performance status of persons with multiple sclerosis (PwMS) is a useful tool to optimize healthcare. This concept does not seem to be extensively explored in this population. This study aimed to determine the level of activity of PwMS during weekdays and weekends, and to establish associations between clinical parameters. Forty-one PwMS and 16 healthy persons participated in this study. Their physical activity in real-life conditions was assessed with an accelerometer. For the clinical evaluations, the quality of life, fatigue, gait, and balance were assessed. The level of activity between PwMS for weekdays, weekends, Saturdays, and Sundays was significantly reduced compared with the reference group (*p* = 0.001–0.00001, d = 0.95–1.76). PwMS had a constant level of activity throughout the week, whereas the reference group increased its level of activity on Saturdays (*p* = 0.04, d = 0.69). The level of activity was correlated in descending order with multiple sclerosis disability, body mass index, gait velocity, six-minute walk test, and timed up and go test. This study showed that PwMS had a stable level of activity throughout the week, contrary to healthy persons. It could be necessary to develop programs to facilitate physical activity and participation during the weekdays, but especially during weekends.

## 1. Introduction

Multiple sclerosis (MS) is a chronic and neurodegenerative disease most often affecting young and middle-aged adults with a female predominance (ratio 2:1). The cause of MS is unknown, although it involves genetic susceptibility and environmental exposure. Thus, the main goals of MS treatment are to delay its progression and to improve quality of life by relieving patients’ symptoms [1].

The symptoms of MS are variable, but typically include sensory, cognitive, and motor impairments [1]. The latter has been reported by persons with MS (PwMS) as one of the most impactful on their lives [2]. A recent survey (*n* = 1011) found that 41% of PwMS had walking difficulties and, for 70% of them, walking disorders seem to be the most impacting factor generated by MS [3].

One of the most important determinants to optimize care for PwMS involves quantifying their functional status (i.e., the ability to walk, to perform daily activities, to meet basic self-care needs). Functional status should be evaluated from different perspectives and at different points along the affected person’s disease progression. Indeed, this functional status, especially walking deterioration, has been used as a major criterion to assess the progression of MS (e.g., the Expanded Disability Status Scale (EDSS) developed by Kurtzke in 1983) [4,5].

Functional capacity and performance are other domains of the functional status of PwMS that should also be explored. These concepts are presented in the current International Classification of Functioning Disability and Health (ICF) developed by the World Health Organization in 2001 [6]. Functional capacity reflects what an individual is capable of doing when performing a task in a standard environment; functional performance reflects what individuals do in their daily lives, and how they interact with their environmental [6].

Most objective functional studies about PwMS were conducted in a standard environment reflecting their functional capacity, such as the well-known Timed 25-Foot Walk test (T25FW) [5]. These studies revealed that, globally, PwMS walk slower, taking shorter steps, with increased step width, and spend more of their gait cycle in double support phase than their healthy peers [7,8,9,10,11,12].

However, little is known about functional performance in PwMS. Measures of objective physical activity in a real context started in the late 1990’s in healthy persons based on accelerometry [13,14]. This method was first used ten years later in neurological patients and studies have been dedicated to validating accelerometry measures. First of all, to conduct this validation process, accelerometry was associated with other well-known clinical subjective questionnaires. The accelerometer expressed in count per day was correlated with physical activity level (respectively for the Godin Leisure-Time Exercise Questionnaire (GLTEQ; r = 0.38) and International Physical Activity Questionnaire (IPAQ; r = 0.34)) [15] or the gait function as the EDSS (r = −0.64) [16] and the Multiple Sclerosis Walking Scale-12 (MSWS-12; r = 0.45–0.68) [15,17,18]. More recently, correlations between accelerometry and objective tests were carried out [19]. These studies suggest a better association between accelerometry and the walking mobility domain, as evidenced with the 6-min walk test (6MWT) (r = 0.78) or the timed up and go test (TUG) (r = −0.68), rather than with the physical activity domain, as demonstrated with the GLTEQ (r = 0.15) or IPAQ (r = 0.35) questionnaires [19].

Although these methodological studies have suggested the clinical application of accelerometry to quantify objectively what PwMS do in their real-life context (in the ICF terms: their performance level), to our knowledge, there few studies that have aimed to compare performance levels between PwMS and other populations (i.e., sedentary healthy individuals) [19,20,21]. These studies conducted in equivalent “ambulatory with minimal assistance” MS populations found that the level of activity during the week corresponded to 52–68% of the level found for matched controls. These studies showed results about the level of activity during an entire week. However, the level of participation and thus, activity level, may be different during weekdays and weekends. Indeed, in Western countries, there are well-established variations in physical activity behavior between the two [22]. During weekdays, people are customarily dedicated to professional activities, whereas, during the weekend, people dedicate their time to leisure, sports or other non-professional activities. Therefore, to improve these previous results, the aims of this study were (i) to determine the level of activity of PwMS in their real-life environment during weekdays and weekends by comparing with that of healthy persons, and (ii) to establish which clinical and functional parameters seem better associated with the real-life activity of PwMS. This study hypothesized that (i) PwMS are less physically active in their real-life than healthy peers during weekdays and weekends and (ii) gait capacity evaluations are associated with real-life physical activity.

## 2. Materials and Methods

### 2.1. Study Design

This was a prospective observational study.

### 2.2. Participants

Forty-one PwMS were included in this study. All PwMS met the following inclusion criteria: (i) MS diagnosis regarding the modified McDonald criteria [23]; (ii) all MS types (i.e., RR, Relapsing–Remitting; SP, Secondary Progressive; PP, Primary Progressive); (iii) the level of disability based on the well-known EDSS (range 1–10) must be between 4.0 and 6.5 which means respectively, significant disability, but self-sufficient, and up about some 12 h a day and requires two walking aids, i.e., pair of canes, crutches, etc.; and (iv) able to walk for at least 6 min.

The exclusion criteria were: (i) worsening MS symptoms (i.e., relapse, Uhthoff’s phenomenon or fatigue increase) during the previous 60 days; (ii) history of epilepsy or epileptic seizures; (iii) immunotherapy change in the previous 60 days; (iv) beginning anti-spastic treatment in the previous 30 days; (v) initiation of treatment able to decrease fatigue symptoms in the previous 30 days; (vi) modification of the rehabilitation program during the study; (vii) motor or other neurological deficits that could interfere with their gait.

Sixteen healthy persons were included as a reference (Ref) group. They were the equivalent of the PwMS group in terms of age, gender, height, and weight. They had no motor or neurological deficits that could interfere with their gait.

The study was approved by the French ethics committee (n. 13/405) and written, informed consent was obtained from all participants.

### 2.3. The Physical Activity in Real-Life Condition Measured by Accelerometry

The physical activity in real-life condition was measured with an ActiGraph, model wGT3X (Actigraph corp, Pensacola, FL, USA), consistent with previous research on validating accelerometer output in PwMS [24,25]. The accelerometer was sampled at 30 Hz and values were expressed as number of counts per minute. Participants were instructed to wear the accelerometer on an elastic belt around the waist (i.e., near to the center of displacement of body mass) located above the hip at the non-dominant side [19,26], to wear it for a 7-day period (including a weekend), and to wear it for the whole day from getting out of bed in the morning until getting into bed in the evening. These instructions were summarized in a memo and given to participants. Afterward, the data were retrieved using ActiLife software (Actigraph corp, Pensacola, FL, USA). Further analyses were conducted if the participant wore the accelerometer for at least 10 h a day and at least 3 or more valid days out of the 7-day period [27]. From the maximum number of days recorded, eight discrete parameters were computed for each participant: average values of counts per minute on (i) weekdays, (ii) the weekend, (iii) the Saturday, and (iv) the Sunday; and peak value of counts per minute on (v) weekdays, (vi) the weekend, (vii) the Saturday, and (viii) the Sunday.

### 2.4. Clinical and Functional Measures

The clinical and functional measures were composed of different and validated tests.

Gait evaluation: The standard gait evaluation was carried out in a dedicated room using the GaitRite system (CIR System Inc., Sparta, NJ, USA), an instrumented walkway embedded with pressure sensors sampled at 120 Hz. The active gait recording surface was 6.1 × 0.61 m. After appropriate instructions and familiarization (about 2 trials), participants were asked to walk with their own standard sports shoes or those provided by our laboratory at their self-selected and fastest speed along the walkway. For each participant, a minimum of 15 steps were recorded. The parameter taken into account for this assessment was gait velocity (m·s^−1^) for both self-selected and fast speed conditions.

Six-minute walk test (6MWT): The 6MWT records the maximum distance a person can walk in 6 min and acts as an endurance walking measure [28]. It assesses the submaximal level of functional capacity. This test was conducted in an oval circuit of 28 m long.

Timed Up and Go test (TUG): The TUG quantifies the time required for a person to rise from a chair, walk 3 m, turn around, walk back to the chair, and sit down. This test is used to quantify functional mobility and balance [29].

Health Related Quality of Life (HRQoL): The PERSEPP scale (Perception de la Sclérose en Plaques et de ses Poussées (Perception of multiple sclerosis and its relapses)) was used to evaluate the HRQoL of PwMS [30]. This scale takes into account several aspects of HRQoL distributed across 33 items and includes the perception related to relapse phases. Each item contains 6 response types according to a Likert scale where “0” was “strongly disagree” and “5” was “strongly agree”. The PERSEPP scale was transformed to a range of 0–100 with high values indicating a high level of HRQoL. The PERSEPP scale is validated in the French language and has a good acceptability (non-return rate < 10%), construct validity (Cronbachs’ α > 0.7) and reliability (ICC = 0.72–0.92) [30].

Fatigue: To measure the fatigue level of PwMS, the Fatigue Impact Scale was used [31,32]. This scale is validated in the French language [32] and distributed across 40 items, which take into account 4 dimensions related to fatigue: cognitive, physical, social and psychological dimensions. Each item contains 4 response types according to a Likert scale where “1” was “always false” and “4” was “always true”. The total fatigue score was standardized from 0–100 with high values indicating a high degree of fatigue.

### 2.5. Procedures

Participants were recruited for this research program at the Laboratory of Clinical Functional Exploration of Movement at the University Hospital of Besançon (Besançon, France). After providing their written informed consent, participants answered a demographic questionnaire and those with MS were evaluated by a single experienced neurologist to determine the EDSS score, MS type, and the Clinical Global Impression (CGI) in relation with the severity of the disease (range from 1 (Normal) to 7 (Among the most extremely ill patients)) [33]. Afterward, the Fatigue and HRQoL questionnaires were completed by PwMS and the functional assessments (i.e., Gait, 6MWT and TUG) described in the Section 2.4 were performed for all participants. To finish, the accelerometer was given to all participants to be worn for seven consecutive days, as described in Section 2.3. Participants returned the accelerometers after the evaluations.

### 2.6. Statistical analysis

Data management and analyses were performed using Statistica version 10 (StatSoft, USA). The results were expressed as mean and Standard Deviation (SD). PwMS and Ref groups were compared in terms of accelerometry, clinical and functional parameters using an independent Student’s *t*-test. Comparisons from ordinal and nominative data were carried out with a Chi-square test. A one-way analysis of variance for repeated measurements was used to compare accelerometry among weekdays, Saturday and Sunday conditions for PwMS and Ref groups. Tukey post-hoc tests were performed when significant effects existed. Afterward, in order to verify the relationship between clinical and functional parameters and the level of real-life activity, Pearson’s correlation tests were conducted. For all analyses, the level of significance was set at 0.05. Finally, effect sizes (d) as well as the coefficient of correlations (r) were calculated to evaluate respectively if differences or associations observed corresponded to important clinical effects [34]. Effect sizes of 0.2, 0.5, and 0.8 were regarded as small, medium, and large degrees of differences, respectively.

## 3. Results

Table 1 shows the participants’ characteristics. A total of 57 participants (PwMS = 41 and Ref = 16) respected all inclusion/exclusion criteria as well as the accelerometer wear instructions. No significant difference was found between PwMS and Ref groups in terms of age, gender, height, body mass, body mass index (BMI), and employment. PwMS had a mean (SD) EDSS of 5.1 (1.1), corresponding to an “Ambulatory without aid or rest for about 200 m”; a disease duration of 14 (10) years, and a CGI of 3.6 (1) corresponding to a “Moderately ill”. As expected, PwMS were significantly different for all functional capacity evaluations (i.e., Gait velocity, 6MWT, TUG), as well as for the perception of their fatigue.

Regarding the accelerometer wear time (Table 2), a significant difference was found between PwMS and Ref groups with effect size indicating medium practical differences for the weekdays (average wear time per day for weekdays: PwMS, 12.88 h vs. Ref, 13.80 h; *p* = 0.02, d = 0.67). Although no significant differences were found for accelerometer wear time for the weekend or separated Saturday and Sunday, a trend was observed for the weekend and Saturday in particular (Table 2).

**Figure 1:**
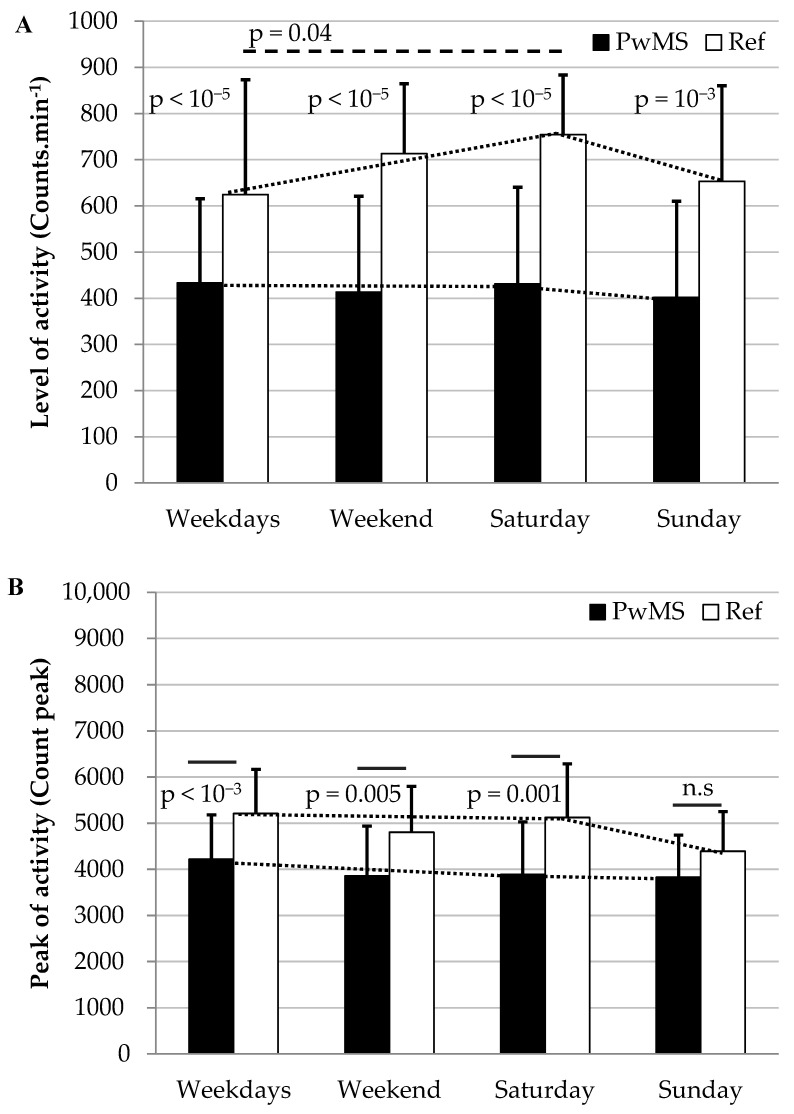
The level of physical activity (mean counts·min^−1^ (**A**) and peak (**B**)) on the weekdays, weekend, Saturday and Sunday and comparisons between MS and Reference groups. PwMS had a monotone level of activity throughout the week, whereas the Ref group had an improvement of activity on Saturday compared with those on weekdays. Abbreviations: PwMS, Persons with Multiple Sclerosis; Ref, Reference group; n.s, not significant.

Table 3 shows the level of activity between groups based on the average number of counts per minute and the peak of counts per minute for weekdays, the weekend, the Saturday and the Sunday. The PwMS group had an average number of counts per minute that was significantly smaller than the Ref group (*p* = 0.001–0.00001, d = 0.95–1.76). These differences corresponded to 57–69% of the values found for the Ref group. Moreover, the PwMS group had a constant level of activity throughout the week, whereas the Ref group increased its level of activity on the Saturday and then decreased it on the Sunday (*p* = 0.04, d = 0.69) (Figure 1A).

Considering the peak of activity (count max), significant differences with moderate to large effect sizes were found between groups for weekdays, the weekend and the Saturday (*p* = 0.005–0.001, d = 0.9–1.08) (Table 3 and Figure 1B). No significant difference was found for the Sunday. Values of peak of activity for the PwMS group corresponded to 76–87% of the values found for the Ref group.

To finish, the level of activity was significant correlated with clinical and functional measures with small to medium effect sizes. In descending order, the correlated parameters were as follows: EDSS, BMI, gait velocity at fast condition, 6MWT, TUG, gait velocity at self-selected condition, and age (Table 4). As expected, all significant correlations were in a clinical sense. For example, as disease severity improved (EDSS), spontaneous physical activity diminished.

## 4. Discussion

As the functional capacity status of ambulatory PwMS has been extensively described in the literature, this study aimed to objectively explore the functional performance status of PwMS and then to identify the associations between this last and assessments that could be done in a clinical or a gait laboratory context.

A first meta-analysis study (13 published articles) found that the level of physical activity of PwMS was largely lower than those found in healthy peers. Based on self-reported measures containing items related to physical activity, mobility limitations, disability, and community participation, PwMS were on average 0.96 SD less active than healthy controls [35].

Recent studies based on objective accelerometer measures confirm this previous result finding that PwMS are still less physically active in their real-life environment than their healthy peers. Moreover, the level of physical activity seems associated with the clinical course and disability status of MS [36,37,38]. In our study, we confirm and complement these previous results (i.e., difference with healthy peers and association with clinical course of MS).

Sandroff et al. found that the level of real-life activity of PmMS with a “Mild disability” level (patient determined disease steps (PDDS): 1) was 0.66 SD lower compared with their healthy peers [21]. From the same research group and for an equivalent study design, Weikert et al. found that the level of real-life activity of PwMS with a “Moderate disability (PDDS: 2) was 1.02 SD lower than their healthy peers [19]. In our results, the differences was comprised between 0.95 SD for weekdays and 1.76 SD for the Saturday (Table 3) for an ambulatory group of PwMS, who had a “moderate-to-severe” disability level (EDSS: 5.1 (1.1)), which corresponds to a score of 5 in the PDDS scale [36]). Moreover, from our clinical and functional capacity parameters tested, the real-life physical activity was best associated with the EDSS, (*r* = −0.58). These results suggest the representativeness of our participants and confirm the influence of disease course on daily-life physical activities.

To our knowledge, previous studies have never described the level of activity during weekdays and the weekend in PwMS. This information could help to better understand their behavior and improve health care. Besides the significant statistical differences between groups, the magnitude of these difference was higher during the weekend (1.57 SD) than the weekdays (0.95 SD) (Table 3). Moreover, contrary to healthy peers who increased their level of activity on Saturdays, PwMS had a constant activity level throughout the week (Figure 1). This may probably have an impact on societal participation of PwMS. As far as we know, until now there is no study about this topic which could make comparisons, but Levin et al. found an association between the social network structure and PwMS’s physical function [39]. Further studies are required to determine if this behavior involves a PwMS’s coping mechanism to save energy (i.e., performance under their maximal capacity level) or a limitation in performance and in this case, PwMS are doing the best that they can during the week (i.e., performance near to the maximal capacity level).

When we consider the peak of activity, which may be an indicator if participants do vigorous physical activity [40], differences between groups were smaller than those found for average values (0.91 SD). On the Sunday, no significant difference was found at all between groups (0.63 SD) (Table 3). For the Sunday, this non-significant difference seems likely to be associated with the reduction of the level of a healthy person’s activity rather than the increase of PwMS’s activity level, since they presented a monotonous activity pattern throughout the week (Figure 1).

Regarding the relationship between PwMS’ real-life activity and their clinical and functional parameters assessed in a standard condition, some values as the EDSS, BMI, age, gait velocity and TUG can testify about a poor performance of the PwMS on daily-life activities. This result was in line a recent study [41]. This information could help clinician’s decision making and the adaptation of patient their health care. Indeed, the substantially sedentary existence of ambulatory PwMS is a challenge in terms of rehabilitation approaches for patients and clinicians since healthy persons (as those compared in this study) already have a sedentary physical activity behavior. A recent study found that only 47.4% of healthy persons meet guidelines recommended by the Word Health Organization and the American College of Sport Medicine in terms of exercise practice [37]. In phase with a previous guideline [42], a recent study gave recommendations for physical activities in PwMS: taking into account comorbidities and symptom fluctuations, healthcare providers should encourage at least 150 min/week of exercise and/or a lifestyle physical activity [43].

Beyond the results of our study, some studies have been shown in PwMS a positive effects of non-pharmacological interventions but not for pharmacological ones [44]. A non-pharmacological example involves studies using Internet interventions based on socio-cognitive theory for increasing physical activity behavior in PwMS. Positive changes in the level of physical activity based on self-reported [45] and objective accelerometer measurements [46] were found with a respective difference of 0.72 and 0.68 SDs. Other medias was also tested to promote physical activity, but with small significant effects [47]. Regarding the effect of these interventions, they could contribute to aligning the level of physical activity with those found in healthy persons during the weekdays, but the level of activity during the weekend may still limited. Politically, urban and environmental planning (e.g., adapted trekking routes, bike lanes) may encourage PwMS to engage in sport or leisure activities on weekends, contributing to their general health.

### Limitations

This study provides a contribution to the knowledge of the objective level of physical activity in real life of PwMS using accelerometry. However, this prospective observational study presents several limitations. The first limitation concerns the wear time of accelerometers by PwMS. During weekdays, PwMS wore accelerometers on average one hour less than their healthy peers. This suggests that PwMS take longer to prepare for their day, take longer in the shower, forget to use accelerometers, or demonstrate a combination of all these factors. Despite the detailed instructions given to the participants explaining how to use the accelerometer and encouraging its use, another medium might improve adherence for this kind of evaluation, such as a telephone call in the week. This was the reason we opted to express values at counts per minute rather than the total number of counts per day, which seems a more intuitive parameter. Second, although the evaluation over seven days was suggested as a good compromise between the representativeness and feasibility of accelerometer measures, further assessment days would improve the wear time and weekend results. Third, it is important to note that our analysis was done from a physical point of view and does not take into account the physiological demands of both groups. For example, for a same peak of count, PwMS may spend much more energy in accomplishing a task than their healthy peers. Some studies have been trying to determine the level of physical activity from a physiological point of view in terms of time spent performing moderate-to-vigorous physical activity per day by accelerometry. The challenge of this approach seems to determine the cut-off points related to these different intensity categories. At the moment, few studies have been dedicated to determining these cut-off points, which seem variable for both healthy [48] and MS populations (i.e., different cut-off points depending on disability levels) [49]. To finish, these results correspond to those obtained in a relatively small French population. Further studies with a larger sample size would improve results by stratifying participants in consistent categories as was previous done in an observational studies (e.g., rural vs urban, employed vs not employed, intellectual vs manual activities, woman vs man… [37,50]) and with participants from multiple countries. Such additional data are necessary to confirm our results.

## 5. Conclusions

In conclusion, our study showed that, during the week, PwMS performed less activity than their healthy peers. PwMS had a stable level of activity throughout the week, contrary to healthy persons whose average and peak values increased on Saturdays. Findings from the current study confirm previous results about the magnitude of the level of activity of PwMS. EDSS, BMI, age, as well as gait velocity and balance measured in a standard condition (functional capacity status) were associated with a poor level of spontaneous physical activity in daily life. It would be worthwhile to develop programs that facilitate physical activity and participation during the weekdays, but especially during weekends.

## Figures and Tables

**Table 1:** Means and (SD) of participants’ characteristics.

Participants’ Characteristics	PwMS (*n* = 41)	Ref (*n* = 16)	*p*
Age (years)	51.3 (12.7)	48.0 (7.6)	0.33
Gender (m/f %)	29.3/70.7	56.3/43. 7	0.11
Height (m)	1.67 (0.09)	1.72 (0.06)	0.07
Body mass (kg)	75.4 (17.1)	72.3 (12.0)	0.50
BMI (kg·m^−2^)	27.1 (6.1)	24.5 (3.1)	0.10
Employment (Employed/Not employed %)	61–39	86–14	0.08
EDSS (0–7)	5.1 (1.1)	NA	NA
Disease duration (years)	13.9 (10.5)	NA	NA
MS type (RR-SP-PP %)	26.8–41.5–31.7	NA	NA
Clinical Global Impression, Severity (1–7)	3.6 (1)	NA	NA
Gait velocity, self-selected (m·s^−1^) *	0.77 (0.4)	1.5 (0.2)	<10^−5^
Gait velocity, fast condition (m·s^−1^) *	1.1 (0.5)	2.3 (0.2)	<10^−5^
6MWT (m) *	267 (151.4)	649 (86.3)	<10^−5^
TUG (s) *	17 (15.6)	5 (0.81)	0.003
Fatigue Impact Scale (0–100) *	59.6 (13.6)	36.8 (11.5)	<10^−5^
HRQoL, PERSEPP Scale (0–100)	59.2 (16.4)	NA	NA

Abbreviations: PwMS, Persons with Multiple Sclerosis; BMI, Body Mass Index; Ref, Reference group; EDSS, Expanded Disability Status Scale; RR, Relapsing–Remitting; SP, Secondary Progressive; PP, Primary Progressive; NA, Non-Applicable; 6MWT, Six-Minute Walk Test; TUG, Timed Up and Go Test; HRQoL, Health Related Quality of Life; PERSEPP, perception of multiple sclerosis and their relapses. *: significant values (<0.05).

**Table 2:** Means and (SD) of participants’ accelerometer wear time on weekdays, weekend and Saturday and Sunday.

Accelerometer Wear Time (h)	PwMS (*n* = 41)	Ref (*n* = 16)	Difference (%) PwMS/Ref	d	*p*
Weekdays *	12.88 (1.44)	13.8 (1.18)	−7.14	0.67	0.02
Weekend	12.78 (2.07)	14.08 (2.26)	−10.17	0.61	0.05
Saturday	13.22 (2.02)	14.53 (2.24)	−9.91	0.62	0.06
Sunday	12.78 (2.33)	14.06 (2.60)	−10.02	0.53	0.12

*: significant values (<0.05); Abbreviations: PwMS, Persons with Multiple Sclerosis; Ref, Reference group.

**Table 3:** Comparisons between MS and the Reference groups for the level of physical activity (Mean and SD for counts·min^−1^ and peak) on the weekdays, weekend, Saturday and Sunday.

Accelerometer Parameters	PwMS (*n* = 41)	Ref (*n* = 16)	d	*p*
Counts·min^−1^ weekdays *	433.46 (181.81)	624.33 (248.83)	0.95	<10^−5^
Counts·min^−1^ weekend *	413.34 (207.82)	713.00 (151.57)	1.57	<10^−5^
Counts·min^−1^ Saturday *	431.20 (209.26)	754.19 (129.12)	1.76	<10^−5^
Counts·min^−1^ Sunday *	402.16 (207.72)	653.18 (207.1)	1.21	10^−3^
Counts peak weekdays *	4218.36 (958.75)	5207.23 (959.02)	1.03	10^−3^
Counts peak weekend *	3856.88 (1077.22)	4802.90 (995.32)	0.90	0.005
Counts peak Saturday *	3888.16 (1137.93)	5123.52 (1158.99)	1.08	0.001
Counts peak Sunday	3829.94 (911.73)	4390.61 (861.66)	0.63	0.07

*: significant values (<0.05); Abbreviations: PwMS, Persons with Multiple Sclerosis; Ref, Reference group.

**Table 4:** Correlations among the level of physical activity (counts·min^−1^) and clinical and functional parameters for PwMS and Ref groups.

	Level of Physical Activity			
	PwMS		Ref	
	**Weekdays**	**Weekend**	**Weekdays**	**Weekend**
**EDSS**	−0.58 *	−0.51 *	NA	NA
**BMI**	−0.51 *	−0.25	−0.53 *	−0.30
**Gait velocity fast condition**	0.48 *	0.35 *	−0.03	0.19
**6MWT**	0.48 *	0.46 *	−0.09	−0.19
**TUG**	−0.48 *	−0.48 *	−0.56 *	−0.25
**Gait velocity self-selected**	0.43 *	0.37 *	0.13	0.12
**Age**	−0.39 *	−0.19	0.03	0.09
**Disease duration**	−0.21	−0.19	NA	NA
**PERSEPP Scale**	−0.19	−0.15	NA	NA
**Fatigue Impact Scale**	−0.02	0.04	NA	NA

*: significant values (<0.05); Abbreviations: PwMS, Persons with Multiple Sclerosis; Ref, Reference group; EDSS, Expanded Disability Status Scale; BMI, Body Mass Index; 6MWT, Six-Minute Walk Test; TUG, Timed Up and Go Test; PERSEPP, perception of multiple sclerosis and their relapses; NA, Non-Applicable.

## Data Availability

The data presented in this study are available on request from the corresponding author.
